# Extraction and immunomodulatory effects of acid *Lagenaria siceraria* (Molina) Standl. Polysaccharide on chickens

**DOI:** 10.1016/j.psj.2024.104113

**Published:** 2024-07-31

**Authors:** En Zhou, Saifuding Abula, Alimujiang abulizi, Guangyan He, Peng Huang, Mutailipu Maimaiti, Dandan Liu, Zhanhai Mai, Shiqi Dong, Adelijiang Wusiman

**Affiliations:** ⁎ College of Veterinary Medicine, Xinjiang Agricultural University, Urumqi 830052, China; † Xinjiang Key Laboratory of New Drug Study and Creation for Herbivorous Animals, Urumqi 830052, China; ‡ College of Veterinary Medicine, Southwest University, Chongqing 402460, China; § Animal Husbandry and Veterinary Station, Shufu County Bureau of Agriculture and Rural Affairs, Kashgar 844100, China

**Keywords:** *Lagenaria siceraria* (Molina) Standl. polysaccharide, H9N2, Vaccine adjuvants

## Abstract

Herbal polysaccharides are extensively studied as vaccine adjuvants due to their safety and potent immunoenhancing activity. This study aimed to analyze the structure of *Lagenaria siceraria* (**Molina**) Standl polysaccharide (**LSP50**) and investigate its adjuvant activity for the H9N2 vaccine in broiler chickens. Structural analysis revealed that LSP50 primarily consisted of rhamnose, arabinose, xylose, mannose, glucose, and galactose with molar ratios of 23.12: 12.28: 10.87: 8.26: 2.64: 22.82 respectively. The adjuvant activity of LSP50 was evaluated, which showing significant enhancements compared to the H9N2 group. Parameters including the immune organ index, H9N2 specific IgG level, cytokines contents (IFN-γ, IL-2, IL-4, and IL-5), and the proportion of CD3e^+^CD8aT^+^cells were significantly increased in the LSP50 group (*P* < 0.05). Additionally, sequencing results showed that LSP50 modulates the immune response by regulating PLA2G12B and PTGDS genes involved in the arachidonic acid pathway. These findings were further validated through qPCR analysis to affirm the reliability of the sequencing data. In conclusion, our results demonstrate that LSP50 exhibits potent adjuvant activity, enhancing both cellular and humoral immunity.

## INTRODUCTION

The H9N2 avian influenza virus (**AIV**) represents a significant subtype within the influenza A virus, exhibiting a broad epidemiological presence and a complex spectrum, making it the predominant subtype of avian influenza in China (Lao et al., 2022; Sajid et al., 2023). Effective immune response to the H9N2 vaccine requires robust humoral and cellular immunity for proliferation (Gan et al., 2019; Du et al., 2023; Yang et al., 2023). Consequently, adjuvants capable of inducing combined humoral and cellular immune responses are crucial for defense and control against H9N2. Vaccine adjuvants play an essential role in enhancing immune responses to vaccines (Liu et al., 2020; Yang et al., 2023), not only by boosting their immunogenicity but also by mitigating potential adverse effects (Feng et al., 2022; Tan et al., 2024). Plant polysaccharides have emerged as a promising adjuvant, demonstrating immunomodulatory properties that augment both cellular and humoral immunity (Sun et al., 2023b). Numerous studies have demonstrated that diverse polysaccharides elicit distinct immune responses, with some inducing mixed immunity while others eliciting singular immune responses. For instance, Zhao et al found that RAMPStp and RAMPS60c significantly promote lymphocyte proliferation, improve antibody titers, and increase the proportion of CD4^+^ and CD8^+^ T cells, making them highly effective immune enhancers (Zhao et al., 2016; Iftikhar et al., 2023). Similarly, Xue et al discovered that the combined inoculation of APS and Newcastle disease live vaccine in laying hens effectively reduces the lethality of the live vaccine on embryos, promotes the growth and development of chickens, and improves overall immunity to Newcastle disease infection (Xue et al., 2020). Furthermore, Yang et al revealed that injecting a dose of 12.5 to 25 mg/kg of (**PTFP**) in chickens vaccinated with the Newcastle disease vaccine resulted in the best immune response The polysaccharide enhanced lymphocyte proliferation, serum antibody titer, and serum IFN-γ level. These findings highlight the potential of PTFP as an immune adjuvant for regulating cellular and humoral immunity (Yang et al., 2019). Shang et al. found that (**TPPPS**), a natural medicinal plant, possesses the ability to inhibit the H9N2 cell action mechanism and prevent virus particles from invading host cells. This provides a theoretical basis for TPPPS as a vaccine adjuvant and is expected to play an important role in preventing related diseases (Shang et al., 2020). Plant polysaccharides hold promise as adjuvants in poultry vaccines, effectively preventing avian disease and enhancing immune response.

*Lagenaria siceraria* (**Molina**) Standl. belongs to Cucurbitaceae family and is a traditional medicine commonly used in Xinjiang and its surrounding areas. It possesses anti-inflammatory, immunomodulatory and antioxidant activities (Saeed et al., 2022). Ghosh et al. extracted polysaccharides from Lagenaria *siceraria* (Molina) Standl. and discovered that they have a molecular weight of 78,000 Da. Structural fragments found in *Lagenaria siceraria* (Molina) Standl. polysaccharides include 1,4-linked α-D-galacturonic acid, 1,2-linked 3-O-acetylmethyl-α-D-galacturonic acid, and 1,4-linked β-D-galacturonic acid. (Ghosh et al., 2009). Deshpande et al. administered *Lagenaria siceraria* (Molina) Standl in doses of 100,200, and 500 mg/kg to rats. The results showed that the fruit could significantly reduce the delayed hypersensitivity of rats, and increase the number of primary antibody titers, secondary antibody titers, white blood cells and lymphocytes. Therefore, this study suggests that this component has an immunomodulatory effect (Deshpande et al., 2008). In our laboratory, we have discovered that LSP50, obtained through 50% alcohol precipitation of *Lagenaria siceraria* (Molina) Standl. Polysaccharides (LSP50), can serve as a Newcastle disease virus (**NDV**) adjuvant to significantly enhance haemagglutination titre and antibody levels while inducing both humoral and cellular immunity in chickens (Wusiman et al., 2022b). We hypothesize that the use of LSP50 could serve as an adjuvant for H9N2 vaccines, enhancing the production of antibodies against H9N2 in chickens and thereby augmenting both cellular and humoral immunity.

Therefore, this study evaluated the immune effect and safety of LSP50 by measuring immune organ index, serum H9N2 specific antibody, serum cytokines, and histopathological sections, and then analyzed the biological function and molecular mechanism of LSP50 on immunity by Illumina sequencing.

## MATERIALS AND METHODS

### Prepared and Characterized of LSP50

The polysaccharide extraction and purification process were conducted as previously described (Wusiman et al., 2022b; Iozon et al., 2023). following this, LSP50 was obtained through 50% alcohol precipitated and the polysaccharides within the main peak of the elution curve were isolated for subsequent analysis. The content of fractions containing polysaccharides, proteins, and glucuronic acid in the collected LSP50 samples was determined using the phenol-sulphuric acid method, BCA kit, and carbazole sulphate method respectively. Additionally, a portion of the of LSP50 samples underwent acid hydrolysis and PMP derivatization followed by HPLC analysis to determine their monosaccharide composition. Furthermore, another portion of LSP50 was sent to Nanjing Yuanjicong New Material Technology Co. for infrared spectroscopy testing.

### Animal Immunization

All experimental procedures were approved by the Animal Welfare Ethics Committee of Xinjiang Agricultural University (Approval No. 2022016). The 1-day-old chickens were acclimatized for 7 d without receiving any drug injections. Subsequently, they were randomly divided into four groups, each consisting of 20 animal: LSP50/H9N2, Alum/H9N2, H9N2, and PBS. Each chick received a 0.3 mL intramuscular injection of vaccine solution. The first immunization was administered on d 20 followed by a second immunization after a 14 d interval. Chickens were euthanized and samples were collected on d 7, 14, and 21 after the second dose, with 5 animals sampled at each time point and 2 replicates identical sample.

### Quantification of Immune Organ Indices

At 7, 14, and 21 d after the second immunization, five randomly selected chickens were euthanized, and their spleen, thymus and bursa were dissected, and their weights were recorded. Immune organ indices were calculated using the formula immune organ index = (weight of immune organ/body weight) × 100%.

### Quantification of H9N2-Specific Antibodies and Cytokines in Serum

At 7, 14, and 21 d after the second immunization, 5 chickens from each group were randomly selected for cardiac blood collection (2 mL). The blood sampled were centrifuged at 3,000 r/min for 10 min to obtain serum. The levels of H9N2-specific IgG and IgA antibodies were quantified using enzyme immunoassay on d 7, 14, and 21. Additionally, the secretion levels of IFN-γ, IL-12, IL-4, IL-5, IL-10in serum were measured on d 21 using ELISA kits (Shanghai Kexing Trading Co., Ltd).

### Histopathological Analysis

At 21 d post the second immunization, the spleen, thymus, bursa of Fabricius, cecal tonsils, and duodenum were collected from the chickens. The tissues were fixed in 4% paraformaldehyde for 24 h and subsequently sent to Wuhan Xavier Biotechnology Co. China for histological examination using hematoxylin and eosin staining.

### Flow Cytometry Detection of T Splenic Lymphocyte Differentiation

At 21 d post the second immunization, the spleen was aseptically collected and homogenized to prepare a single-cell suspension. Erythrocytes were eliminated by adding red blood cell lysis buffer, and the cells were subsequently washed twice with PBS buffer. The cells were then labeled with anti-CD3e-FITC, anti-CD8a-PE (France abcam Co., Ltd), followed by incubation at 4°C in darkness for 45 min. After 2 times washing with PBS buffer, the cells were resuspended and subjected to flow cytometry analysis.

### Duodenal RNA Extraction and 16S mRNA Sequencing Analysis

At 21 d post the second immunization, chicken duodenum samples were collected for RNA extraction using Trizol reagent. The extracted total RNA was sent to Nomi Metabolism for detection. Simultaneously, reverse transcription of the total RNA into cDNA was performed using a reverse transcription kit (Vazyme Biotech Co., Ltd), and the resulting cDNA was stored at -80 °C till further analysis.

The qPCR reaction conditions included pre-denaturation at 95 °C for 30 s, followed by a cycling reaction at 95 °C for 10 s and 60 °C for 30 s, repeated for a total of 40 cycles. A melting curve was generated with temperature steps of 95 °C for 15 s, followed by incubation at 60 °C for 60 s and another step at 95 °C for an additional 15 s. Three replicates were performed per sample, and the relative mRNA expression levels of PLA2G12B, PLA2G2E, HPGDS, EPHX1L, and UGT1A1 genes were determined using the comparative Ct method (2-ΔΔCt), normalized to β-actin expression.

### Primer Design and Synthesis

The chicken mRNA sequences were acquired from the National Center for Biotechnology Information (NCBI), and primer design was conducted using Primer Premier 5 software. The specific primer sequences are presented in Table 1 below.

**Table 1:** Primer's sequence of qPCR.

Gene	→ Primer sequence (5′-3′)	Base number
PTGDS	F:AACAGTGCGAGAAGAGGAAC	20
	R:GTCTGGGGTAGGATGAGGAT	20
PLA2G12B	F:GGGACAGCTTCGAGACGGTTAA	22
	R:TGTAGTGAGGTCGTGGCATTGG	22
PLA2G2E	F:CGTTCCCCACCTTGCCACTTA	21
	R:AAACACTCTGCTGCCTTCTTG	21
HPGDS	F:CTGATTTCTACTGGGATGTGT	21
	R:TGCTGTCTTTGGTCTTTTCT	20
GSTA3	F:TTCTGCACTATGCCAACACAC	21
	R:CACCATCTTCATCCCATCAAT	21
HSD11B1b	F:TGAGGAGGTGGTGAAAGAGG	20
	R:AGAGTAGGGAGCAACGAAGG	20
EPHX1L	F:CACATTTCCATACCACCATT	20
	R:TCAAACACCACATCACCACT	20
UGT1A1	F:AGATGGACAATGCGAAACGA	20
	R:CCAGGAGGAAGGCAAAGACG	20
β-actin	F:ACCGGACTGTTACCAACACC	20
	R:GACTGCTGCTGACACCTTCA	20

### Statistics Analysis

The experimental data were analyzed using IBM SPSS Statistics 24 software, utilizing one-way ANOVA-Duncan multiple comparisons and reporting the results as "X ± SE". Statistical significance was determined at the *P* < 0.05 level, indicating significant differences.

## RESULTS

### LSP50 Isolation and Purification

**Figure 1:**
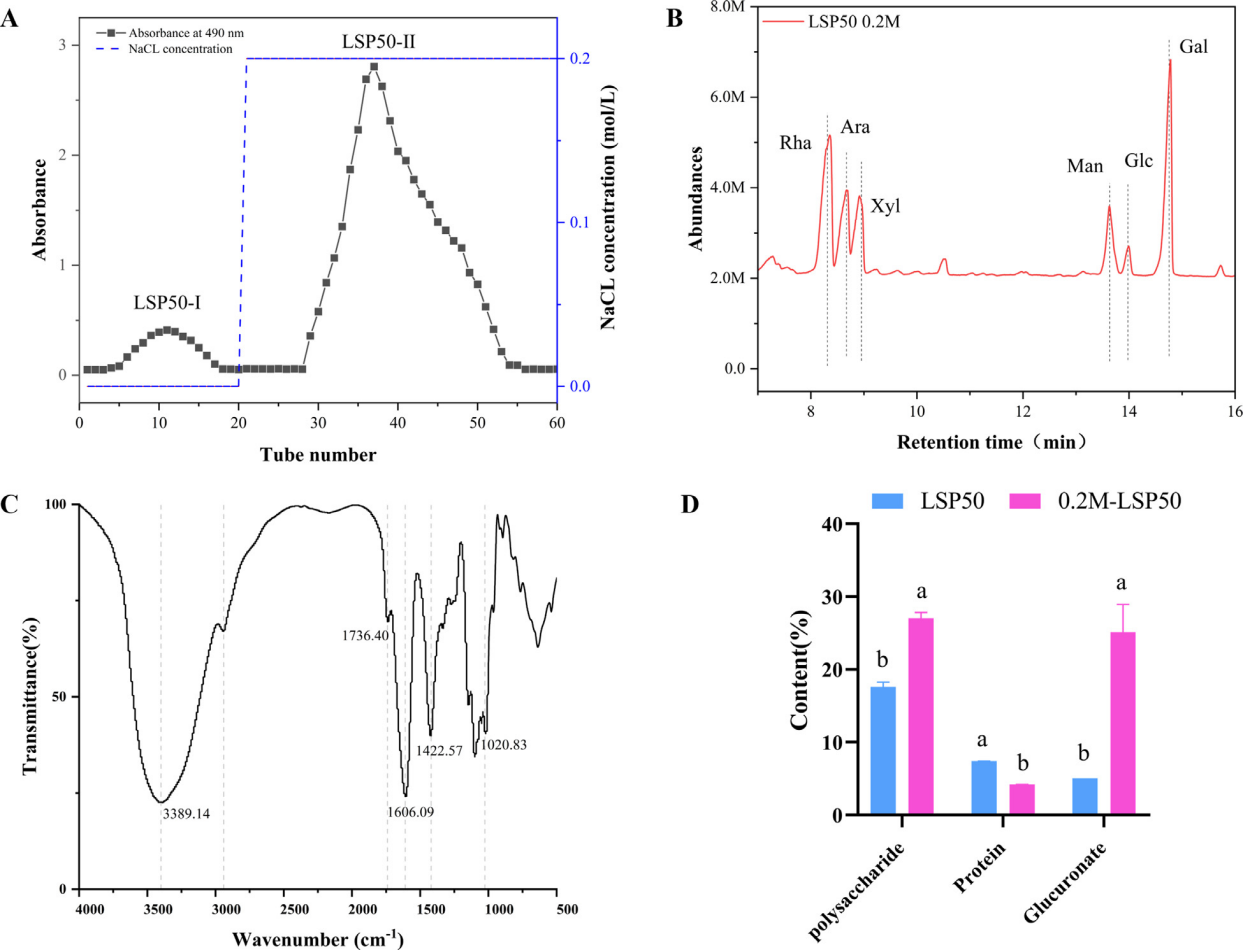
Polysaccharide characterization and quantification. (A) LSP50 elution curve (B) LSP50 monosaccharide fraction (C) LSP50 infrared spectrum (D) Sugar, glucuronic acid and protein content.

**Figure 2:**
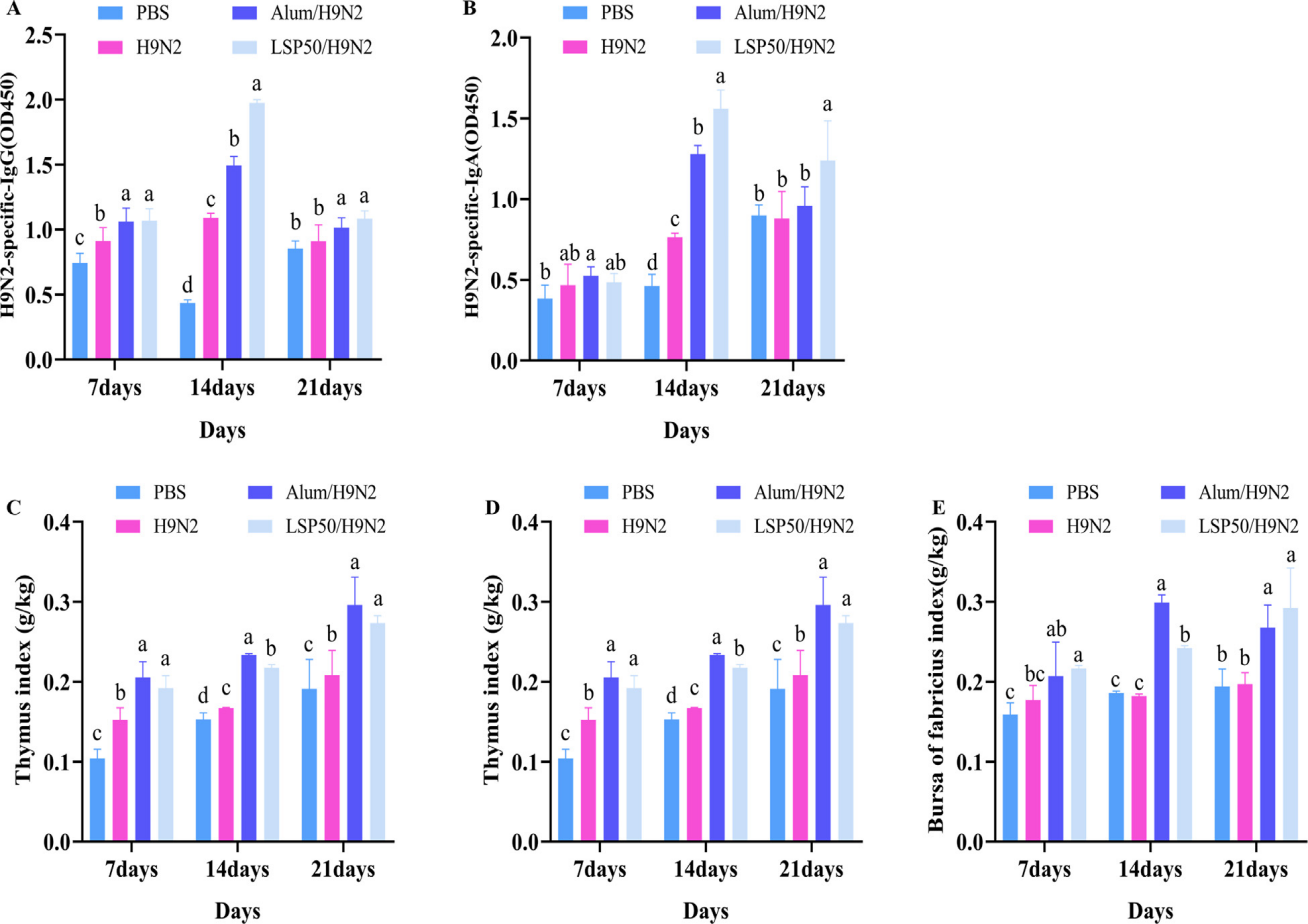
Antibody levels and immune organ index. (A) Specific IgG Antibody. (B) Specific IgG Antibody. (C-E) 7-, 14- and 21-d immune organ index. a-d Superscript different significant difference (*P* < 0.05), n = 5.

The LSP50 was purified by using gradient elution through a DEAE-52 cellulose gel column, as shown in Figure 1A. The result presented in Table 2 and Figure 2 revealed that the monosaccharides were predominantly composed of rhamnose, arabinose, xylose, mannose, glucose and galactose with a molar ratio of 23.12: 12.28: 10.87: 8.26: 2.64: 22.82. The strong absorption peak of the LSP50 at 3,389.14 per cm was attributed to the telescopic vibration of the O-H group of the polysaccharides. Additionally, the absorption peak at 1,736.40 per cm is indicative of the stretching vibration of the C-O or COO- group, suggesting the potential presence of galacturonic acid. (Wang and Guo, 2020). The absorption peak at 1,606.99 per cm corresponds to the C=O asymmetric stretching vibration absorption peak of -COOH, while the absorption peak at 1,422.57 cm-1 represents the -OH absorption peak caused by the C-H varus angular vibration. Moreover, a set of strong absorption peaks at 1,020 is observed. The peak at 83 per cm is arises from the stretching vibration of C-O-C on the glycan ring. This peak serve as a characteristic absorption peak of the pyran ring, indicating the presence of pyranose as well as glyoxalate in the structure of LSP50 (Figure 2C). The contents of polysaccharides, proteins, and glucuronic acid were determined both pre- and postpurification, content of 17.6, 7.4, and 5.05% before purification, respectively, and 27, 4.2, and 25.1% after purification. (Figure 1D).

**Table 2:** Monosaccharide composition content of LSP50.

Monosaccharide type	Monosaccharide content
Rhamnose	23.1%
Arabinose	12.3%
Xylose	10.87%
Mannose	8.26%
Glucose	2.64%
Galactose	22.82%

### Immune Organ Index and Specific Antibody Levels

The levels of H9N2-IgG and H9N2-IgA in the LSP50/H9N2 groups were significantly increased on d 14 and 21 compared to the H9N2 group (*P* < 0.05; Figures 2A and 2B). On d 7, there was non-significant difference (*P* > 0.05) in H9N2-IgA levels between the LSP50/H9N2 group and the H9N2 group, but H9N2-IgG levels in the LSP50/H9N2 and Alum/H9N2 group were significantly higher than those in the H9N2 group (*P* < 0.05). The Figures 2C-2E showed that the spleen, thymus, and bursa indices were significantly higher in the LSP50/H92 and Alum/H9N2 groups compared to the PBS group on d 7, 14, and 21 (*P* < 0.05). On d 7 and d 21, there were no significant differences in spleen, thymus, and bursa indices between LSP50/H9N2and Alum/H9N2 groups.

### Cytokine Levels

**Figure 3:**
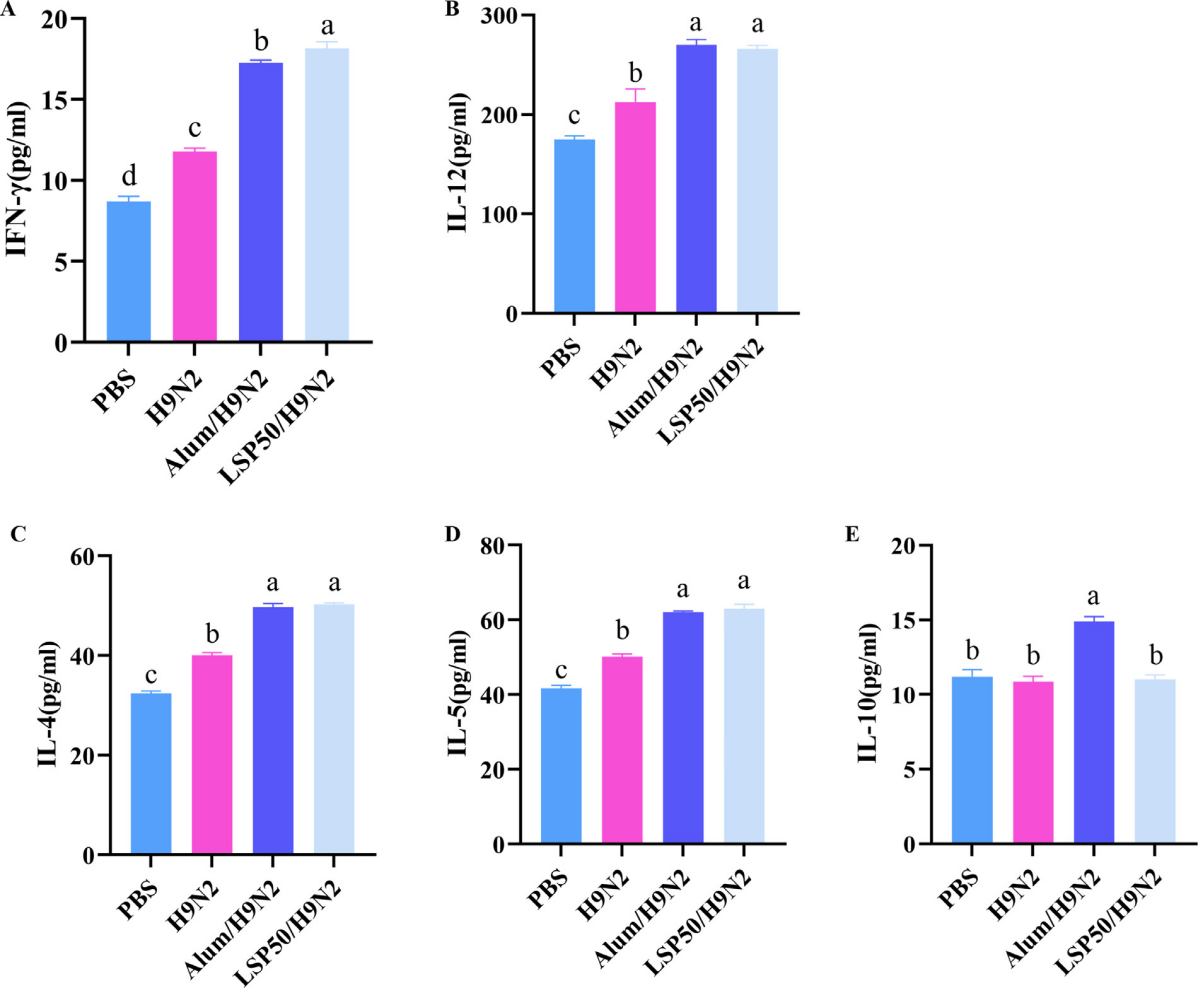
Cytokine expression. (A) Concentration of IFN-γ. (B) Concentration of IL-12. (C) Concentration of IL-4. (D) Concentration of IL-5. (E) Concentration of IL-10. a-d Superscript different significant difference (*P* < 0.05), n = 5.

As illustrated in Figures 3A and 3B, the IL-12 and IFN-γ contents in the LSP50/H9N2, Alum/H9N2, and H9N2 groups were significantly higher than those in the PBS group (*P* < 0.05). Additionally, the IFN-γ content in the LSP50/H9N2 group was significantly higher than that in the Alum/H9N2 and H9N2 groups (*P* < 0.05). However, there was no significant difference between the IL-12 content in the LSP50/H9N2 group and Alum/H9N2 groups (*P* > 0.05). The levels of cytokine in each group are depicted in Figures 3C-3E. The IL-4 and IL-5 contents in the LSP50/H9N2, Alum/H9N2, and H9N2 groups were significantly higher than those in the PBS group (*P* < 0.05). The IL-4 and IL-5 contents in the LSP50/H9N2 group were significantly higher (*P* < 0.05) than H9N2 group. There were non-significant differences (*P* > 0.05) in IL-4 and IL-5 contents between the LSP50/H9N2 and Alum/H9N2 groups. The content of IL-10 in the LSP50/H9N2 group was not significantly different from H9N2 and PBS groups (*P* > 0.05).

### HE Stained Sections

**Figure 4:**
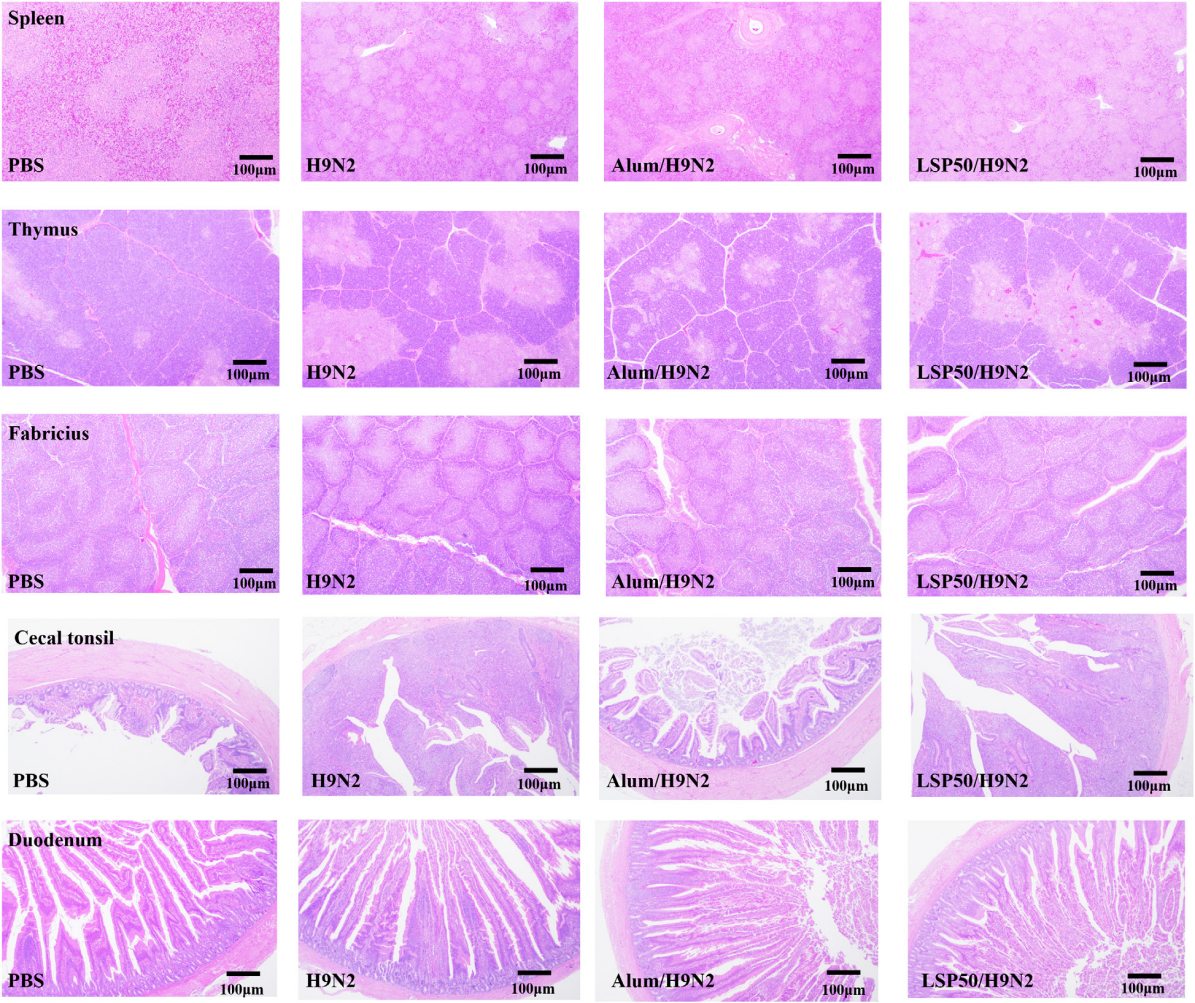
Histological analysis of thymus, spleen, bursa, jejunum, cecum tonsils (100×, HE, scale bar 100 μm).

No lesions or inflammation were observed in the spleen, thymus, bursa, cecum tonsils, and duodenum of the chickens, indicating that the LSP50 vaccine adjuvant has a good safety profile. As depicted in Figure 4, the number of splenic microsomes in the LSP50/H9N2 and Alum/H9N2 groups was significantly higher compared to the PBS group. They exhibited larger size with a clear demarcation between the red and white medulla. Additionally, the LSP50/H9N2 group had a significantly higher number of thymic lobules compared to the PBS group, with an increase in lobule area and the number of thymic microsomes in the medulla increased. Moreover, the number of lymphoid follicles in the mucous membranes of the bursa of the Fabricius was significantly higher in the LSP50/H9N2 group compared to the PBS group. when compared to the PBS group, the lymphoid follicles in the folds of the bursa mucosa of the LSP50/H9N2 group exhibited complete morphology, clear demarcation, increased lymphoid follicle area, increased inter-follicular spacing, and gradual enlargement and lengthening of the lymphoid follicle morphology, with a more regular arrangement of the lymphoid follicles. Furthermore, in the LSP50/H9N2 group, the number of germinal centers at the bottom of the mucosal lamina propria increased, and their outlines became clearer. The mucosal lamina propria of the bursa mucosa in the LSP50/H9N2 group showed a number of cecal tonsillar germinal centers. In comparison to the PBS group, the experimental group exhibited intestinal mucosal epithelial cells with clear outlines, regular morphology, a clear structure of the lamina propria, well-developed intestinal glands, and neatly arranged villi that were denser and longer.

### Activation of T Splenic Lymphocytes

**Figure 5:**
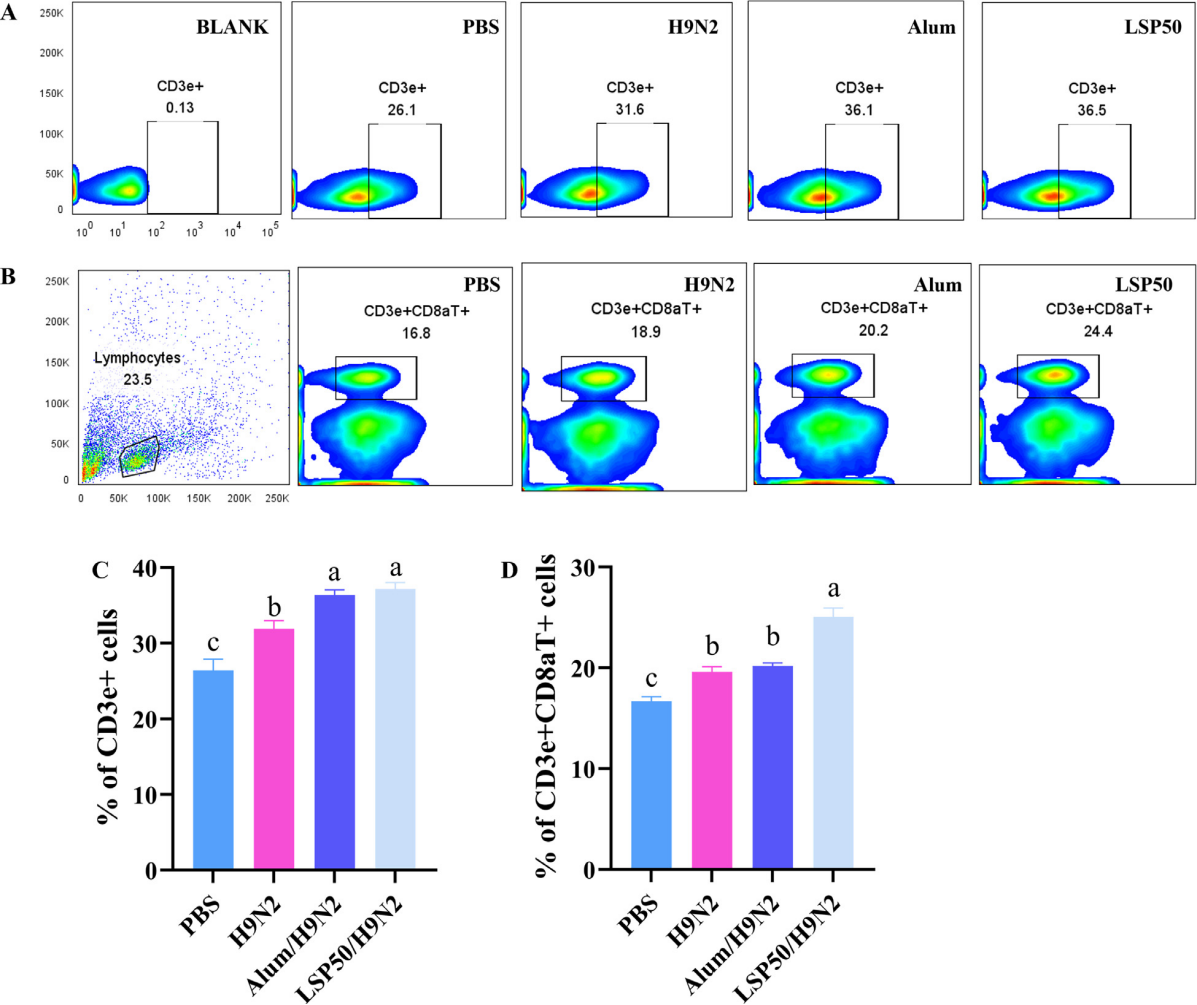
Differentiation of CD3e^+^ and CD3e^+^CD8aT ^+^ cells in spleen. a-d Superscript different significant difference (*P* < 0.05).

Spleens were collected from chickens 21 d after the second immunization, and splenocytes were extracted. As depicted in Figure 5, there was no statistically significant difference observed in the CD3e^+^ cells outcomes between the LSP50/H9N2 group and the Alum/H9N2 group. However, notably higher activation results were observed in both CD3e^+^ and CD3e^+^CD8aT^+^cells populations within the LSP50/H9N2 group compared to those of the H9N2 and PBS groups. These findings suggest that LSP50 has the potential to enhance the T cell-mediated immune responses.

### Overall Genetic Changes

**Figure 6:**
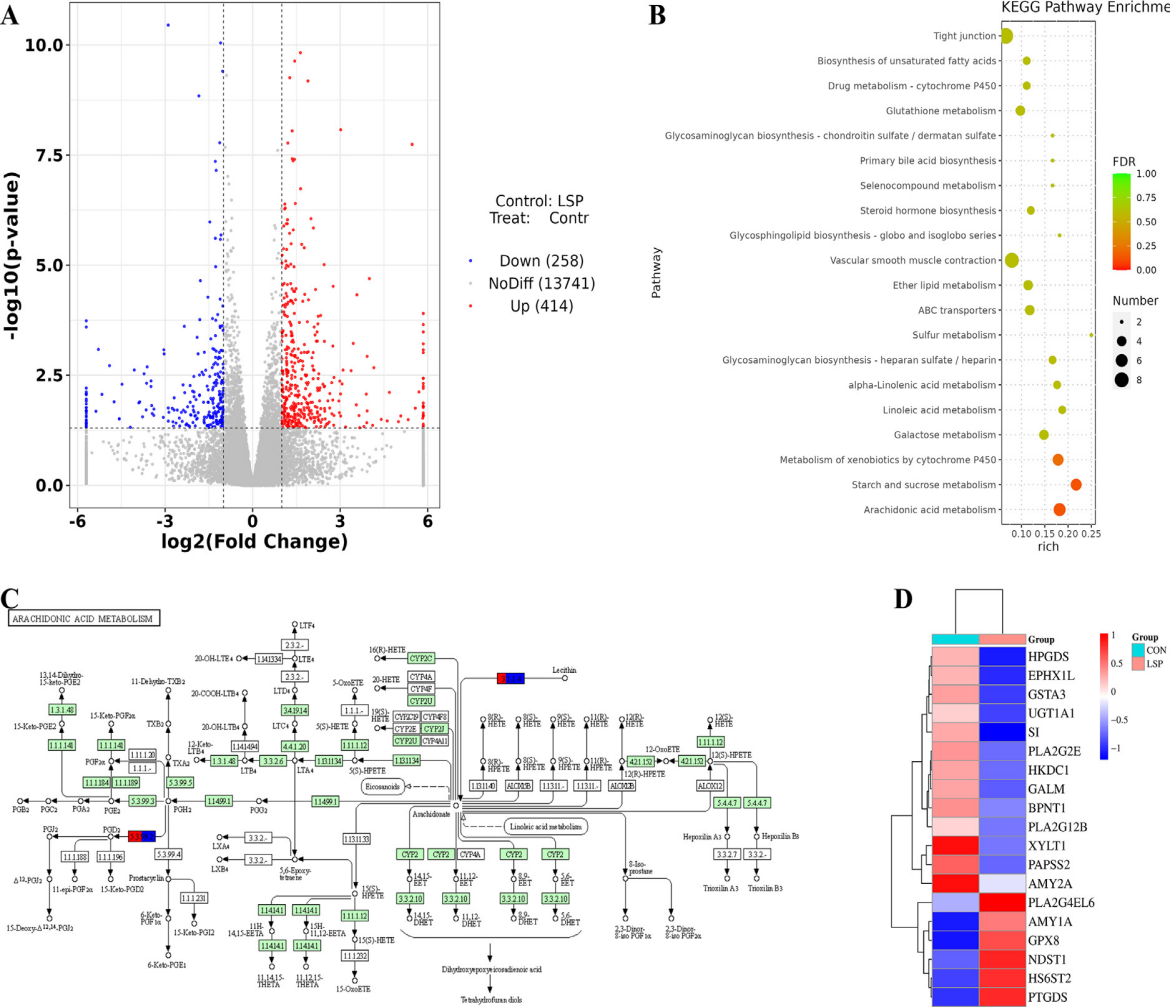
Sequencing results. (A) Differential gene volcano map. (B) KEGG Bubble Chart. (C) Arachidonic acid pathway map. (D) Heat map of key genest.

The differentially expressed genes (**DEG**) in the PBS and LSP50 groups were obtained by analyzing the differential genes based on the data obtained from the gene expression analysis. To explore the effect of LSP50, the results of the gene changes on the 21st d after the second immunization were examined using the PBS group as a control. As seen in Figure 6A, a total of 672 genes exhibited differences, with 414 being up-regulated and 258 being down-regulated. In Figure 6B, Upon KEGG analysis, the 20 most important immune pathway entries were found to be enriched. These pathways include arachidonic acid metabolism, metabolism of xenobiotics by cytochrome P450, and alpha-Linolenic acid metabolism. In the arachidonic acid metabolism pathway, gene expression of PLA2G12B and PLA2G2E was down-regulated, while PTGDS gene expression was up-regulated (Figure 6C). To analyze the LSP50 immunoregulatory pathway, a heat map was created to display changes in gene expression. The results showed that PLA2G12B, PLA2G2E, HPGDS, and UGT1A1 gene expression were downregulated, while PTGDS, GSTA3, and HSD11B1b gene expression was upregulated.

### Gene mRNA Expression

**Figure 7:**
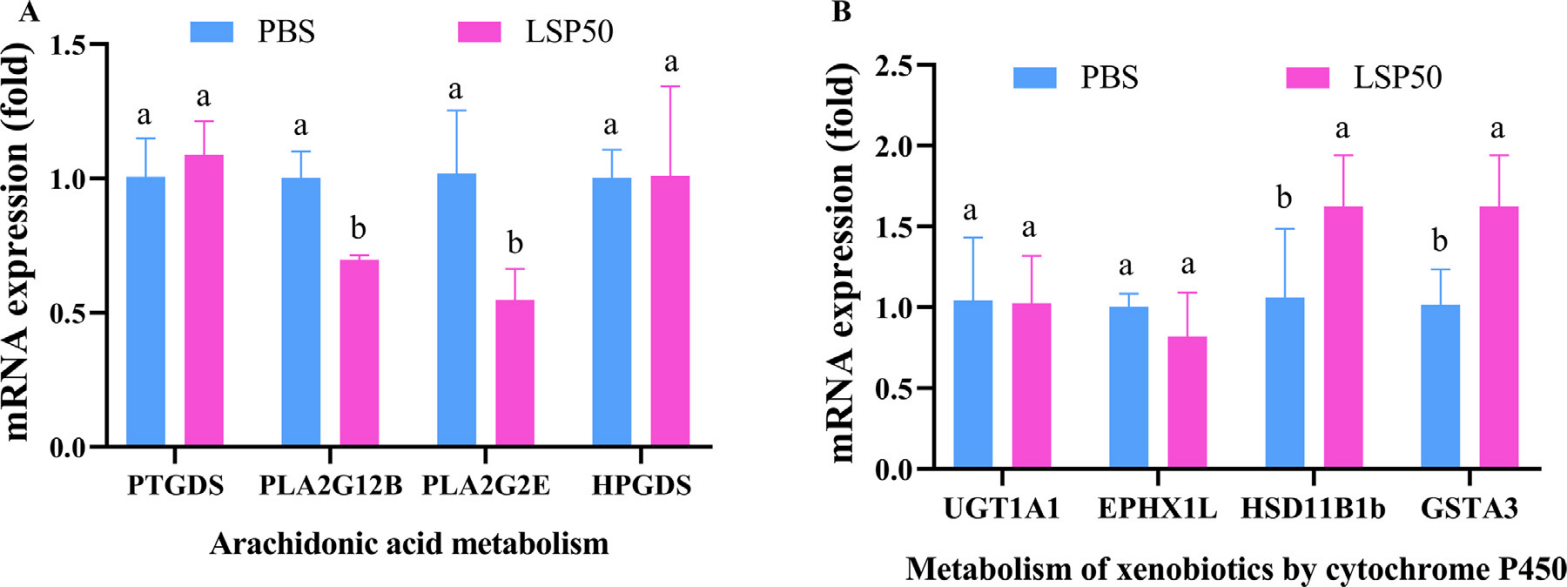
mRNA expression level. (A) PTGDS,PLA2G2E,HPGDS and PLA2G12B gene mRNA expression. (B) GSTA3,EPHX1L,HSD11B1b and UGT1A1 gene mRNA expression. a-d Superscript different significant difference (*P* < 0.05).

In the PBS group, a significant downregulation of PLA2G12B, PLA2G2E, and HPGDS genes was observed compared to the LSP50 group (Figure 7A). Conversely, PTGDS genes were increased, which is consistent with the results shown in Figure 7A, and suggesting the presence of an arachidonic acid metabolism pathway. The expression levels of EPHX1L and UGT1A1 genes were decreased in the PBS group compared to the LSP50 group, while GSTA3 and HSD11B1 genes showed increasing trend. These findings are consistent with the results presented in Figure 6, which suggest the involvement of a metabolism of xenobiotics by cytochrome P450 pathway.

## DISCUSSION

Vaccination represents a crucial strategy for preventing infectious diseases in animals, with adjuvants play a pivotal role in enhancing the immune response of vaccines (Liu et al., 2020; Yang et al., 2023). A well-designed immune adjuvant not only enhances the efficacy of vaccines but also mitigates their potential side effects (Feng et al., 2022; Telli et al., 2022). Herbal polysaccharides possess multifaceted immunostimulatory properties with multiple targets and functions, and numerous studies have corroborated their immune-adjuvant effects (Ren et al., 2019). However, the composition and immunological activity of LSP remain elusive. In this study, polysaccharides were extracted through hydroalcohol precipitation and subsequently purified using a DEAE-52 cellulose gel column to obtain LSP50. Chickens were immunized via intramuscular injection, followed by an assessment of their immunological response. The mechanism underlying immune production was analyzed employing KEGG.

Polysaccharides exhibit diverse biological activities contingent upon their composition, structure, and content (Karimi et al., 2021; Wang et al., 2023). Consequently, determining monosaccharide composition in polysaccharides is of paramount importance for investigating the immunological potential. Zhu conducted a comprehensive investigation on the structural characterization, physicochemical properties, antioxidant and antitumor activities of polysaccharides derived from Polygonum multiflorum. The observed variations in the antitumor activities between neutral and acidic polysaccharides were potentially attributed to differences in monosaccharide composition, molecular weight, glycosidic bonding patterns, and glyoxalate content (Zhu et al., 2017; Hussain et al., 2023). The results depicted in Figure 1 demonstrate that LSP is an acidic polysaccharide primarily composed of monosaccharides, including galactose and glucose. These findings suggest that LSP50 possesses the potential to elicit immune-enhancing effects.

Immunoglobulin is an essential component of the body's disease resistance, with IgA playing a crucial role in mucosal immunity and IgG being the primary antibody produced by humoral immune responses with significant immune effects (Cain et al., 2023; Conrey et al., 2023). our results depicted that LSP50/H9N2 significantly augmented the levels of H9N2-IgA and H9N2-IgG compared to the chickens in the H9N2 group, indicating that LSP50 can elicit both mucosal and humoral immune responses. The spleen, thymus and bursa indices are core markers for assessing immune organ development in chickens; an increase in their mass represents organs immune status (Sun et al., 2023). Our results show that injecting LSP50 into chickens during immunization significantly increases their thymus, spleen and bursa indices. We speculate that some metabolic pathways within LSP50 promote metabolism-mediated enhancement of organismic immunity.

Cytokines play a crucial role in stimulating the immune response following antigen invasion (Zheng et al., 2022). Th1 cells derived from CD4 cells, secrete type 1 cytokines such as IL-2 and IFN-γ, which possess antiviral activity and promote the proliferation of CD8^+^ cells (Ruterbusch et al., 2020; Shang et al., 2024). Similarly, Th2 cells also originate from CD4 cells and produce type 2 cytokines including IL-4, IL-5, and IL-10. These cytokines induce paracrine activity in B cells and stimulate humoral immune responses (Kokubo et al., 2022; Karakurt et al., 2023). These are primarily secreted by Th2-like cells, while IL-4 can also be produced by granulocytes and myeloid cells. It is closely associated with the development of T and B lymphocytes as well as the production of humoral immunity and antibodies (Gärtner et al., 2023). IL-5 is a pleiotropic cytokine that participates in immune responses (Kandikattu et al., 2019). Furthermore, IL-10 acts as an anti-inflammatory cytokine regulating the balance of immune cells within the immune system (Wang et al., 2019). IL-12 promotes Th1 immune response by strongly inducing IFN-γ production in T and NK cells (Glassman et al., 2021). Whereas IFN-γ plays an important role in intrinsic immunity during specific immunity mediated by CD4 Th1 and CD8 cytotoxic T-cells secretion process (Jorgovanovic et al., 2020). Detection of cytokines allows assessment of whether polysaccharides favor Th1 or Th2 cell responses or not. The results obtained from chickens (e.g., Figures 3A-3E) demonstrated that immunization with LSP50 led to increased levels of both Th1-type cytokines (IFN-γ&IL-12) along with elevated levels of Th2-type cytokines (IL-4 & IL-5) compared to PBS group. These findings suggest that LSP50 possesses the ability to induce both cellular-mediated immunity through activation of APCs migration towards lymph nodes.

Histological examination (HE sections) is commonly used to observe pathological changes in tissue samples and evaluate inflammation or other focal points within an organism. No lesions or signs of inflammation were observed in the spleen, thymus, bursa, cecum tonsils, and duodenum of the chickens, as depicted in Figure 4. This indicates that LSP50 exhibits a favorable biosafety profile as an adjuvant. Flow cytometry is a valuable tool for identifying different subsets of T cells (Frangiamone et al., 2022). The spleen plays a crucial role in regulating both innate and adaptive immunity (Bevers et al., 2022). T cells provide immune protection against various diseases, eliminate cancerous cells and may contribute to autoimmune deficiencies. CD3^+^ cells serves as a common surface marker for all types of T cells including helper T cells, regulatory T cells, and killer T cells. Upon recognition of pathogens by MHC II, CD4^+^ T cells produce antibodies that recruit other immune cells to the site of infection, thereby eliminating the pathogen (Chapman et al., 2020). Conversely, CD8aT^+^ cells recognize and eliminate pathogens presented on MHC I (Lees, 2020). Therefore, CD3e^+^ and CD8aT^+^cells serve as important markers for identifying activated T cells (Wusiman et al., 2022a; O'Neil et al., 2023). The results illustrated in Figure 5 demonstrated that LSP50 significantly enhanced activation levels in both CD3e^+^ and CD8aT^+^cells lymphocytes at day 21, suggesting that LSP50 can enhance immunity.

Arachidonic acid (**AA**) is naturally present in the structural phospholipids of cell membranes in the body or stored in immune cell liposomes (Tallima, 2021; Nworah et al., 2022). AA is susceptible to oxidation due to its 4 double bonds, and lead to the production of numerous metabolites that play a crucial role in normal immune system function (Hanna and Hafez, 2018). Phospholipase A2 (**PLA2**) catalyzes the release of AA from membrane phospholipids, which is then converted into PGH2 by cyclooxygenase (**COX**, including COX-1 and COX-2). Subsequently, PGE synthase catalyzes the conversion of PGH2 into PGE2 (Wang et al., 2021). The enzyme phospholipase A2 (**PLA2**) plays a crucial role in the hydrolysis of cell membrane phospholipids to release free fatty acids like AA, which has been demonstrated to contribute significantly to inflammation (Letsiou et al., 2021). PGE2 has been shown to modulate immune response by inhibiting macrophage antigen presentation and T cell immunity (Wang et al., 2022). PLA2G12B belongs to the PLA2 family and PTGDS acts as a PGE2 synthetase. These genes may regulate immune response through their high or low expression levels. In KEGG analysis, down-regulation of PLA2G12B gene suggests potential protection against inflammation and enhancement of immunity, while up-regulation of PTGDS gene indicates regulation of immune response through PGE2 production (Figure 6). qPCR is considered one of the most effective techniques for quantifying mRNA expression levels of different target genes, and employing internal reference genes is commonly used method to ensures the accuracy and standardization of qPCR assays (Divashuk et al., 2022). The heatmap results demonstrated that PLA2G12B, PLA2G2E, HPGDS, EPHX1L, and UGT1A1 were down-regulated in the LSP50 group compared to the PBS group. Conversely, PTGDS, GSTA3, and HSD11B1b were up-regulated in the LSP50 group. These findings are in line with the heatmap results, which further validate the sequencing results.

## CONCLUSIONS

In summary, purified LSP50 is an acidic polysaccharide comprising 27% polysaccharides and 25.1% glyoxylates. LSP50 not only induces the production of antigen-specific levels in chickens but also enhances the expression of Th1 and Th2 cytokines. Furthermore, LSP50 demonstrates favorable biosafety characteristics and exerts its immunological activity through the arachidonic acid pathway. Therefore, it is concluded that LSP50 exhibit efficacy as an H9N2 vaccine adjuvant for the prevention of avian influenza.

## DISCLOSURES

The authors declare no conflicts of interest.

## Appendix Supplementary materials

Image, application 1
