# Supplementary material for: Extraction and immunomodulatory effects of acid Lagenaria siceraria (Molina) Standl. Polysaccharide on chickens
Source: Poult Sci. 2024 Jul 31;103(10):104113. doi: 10.1016/j.psj.2024.104113 (PMC11379659; doi:10.1016/j.psj.2024.104113)
Supplement: Supplementary file 1 [file mmc1.docx]

**Extraction and immunomodulatory effects of acid** ***Lagenaria siceraria* (Molina) Standl. Polysaccharide on chickens**

**SUPPLEMENTAL INFORMATION**

The chickens were acclimatized and reared for 7 days prior to the initial immunization, followed by two subsequent immunizations at 7-day intervals. Dissections were performed at 7, 14, and 21 days after the second immunization to obtain the spleen, thymus, and bursa of chicks for weighing and calculation of immune organ indices. Serum centrifugation was conducted to assess the immune response capacity of chicks by testing for IgA, IgG, and cytokines. Spleens were collected for T-lymphocyte differentiation analysis while RNA extraction from the duodenum was carried out for sequencing and validation purposes.

**
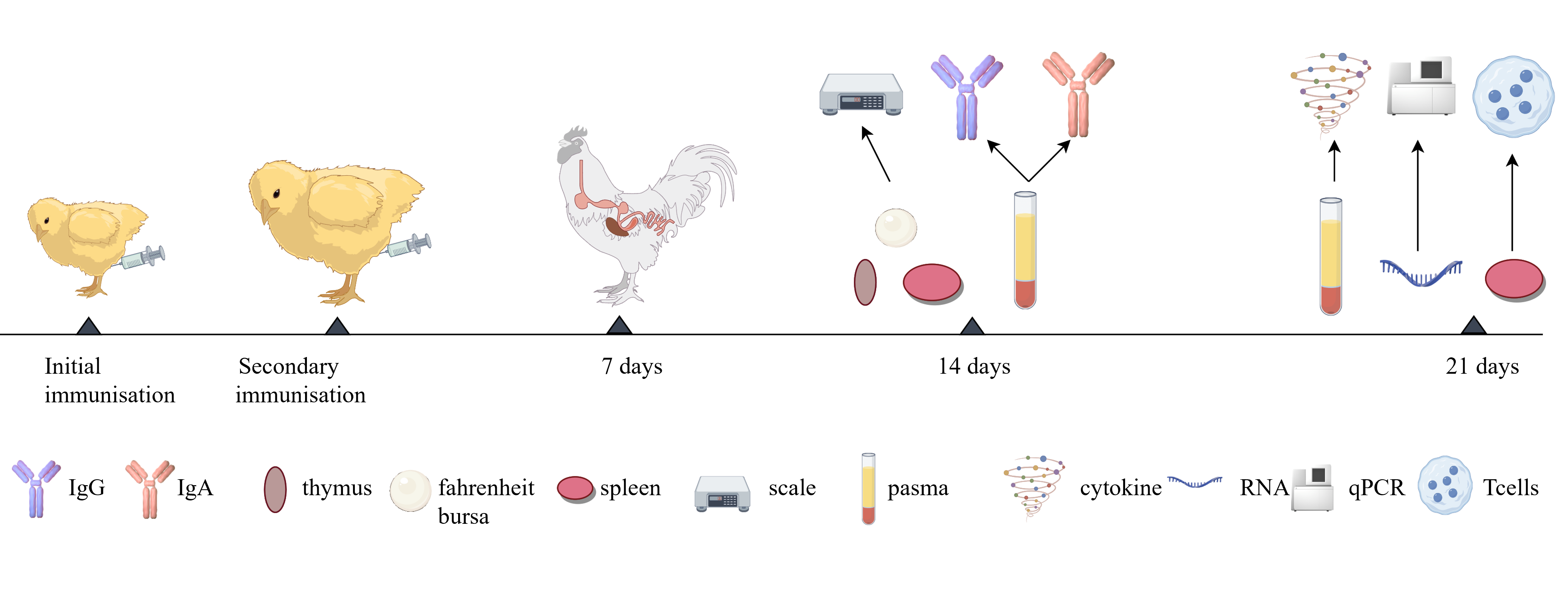
**

**Figure S1 Immunisation flowchart**
